# Exploring the potential of acupuncture as a complementary therapy for post-COVID skin hyperpigmentation: a case report

**DOI:** 10.3389/fmed.2025.1598618

**Published:** 2025-07-09

**Authors:** Yuan Xue, Xingyao Li, Yingying Duan, Qi Gao, Yanhong Zhao, Xili Wu, Zhongheng Wu

**Affiliations:** Department of Traditional Chinese Medicine, The Second Affiliated Hospital of Xi'an Jiaotong University, Xi’an, China

**Keywords:** SARS-CoV-2 coronavirus, COVID-19, acupuncture, case, complementary therapies

## Abstract

**Background:**

Post-COVID sequelae include skin abnormalities with nonspecific characteristics, making disease-specific diagnosis challenging. Conventional treatment primarily relies on modern medical approaches, and the potential of complementary therapies, such as acupuncture, remains underexplored.

**Case report:**

This case describes a patient who developed abnormal skin pigmentation following a COVID-19 infection and was initially diagnosed with five different conditions. After a prolonged period of unsuccessful treatment with conventional medicine, the patient received complementary therapy, leading to significant symptom improvement.

**Conclusion:**

Complementary therapies, such as acupuncture, may prove beneficial in treating skin conditions that lack a definitive diagnosis, as demonstrated in this case.

## Introduction

In early 2020, a viral infection was identified in Wuhan, China, which rapidly affected a large number of individuals. The World Health Organization (WHO) later confirmed that the epidemic was caused by the SARS-CoV-2 coronavirus. By 2023, the virus had infected over 700 million people globally.^1^ The virus persists to this day, continuing to cause significant morbidity and complications. Research has shown that COVID-19 is associated with various skin manifestations, including pseudo-chilblains, urticaria, maculopapular eruptions, other vesicular eruptions, livedo, and necrosis (1).

Acupuncture, a form of traditional Chinese medicine (TCM) and alternative therapy, has been practiced in China for over 2,000 years. In recent decades, its popularity has grown in the United States, with prevalence rates ranging from 0.6 to 1.4% (2). A survey indicated that 6% of participants with skin diseases used alternative therapies, with acupuncture representing 9.3% of these cases (3). Studies suggest that acupuncture can improve outcome indicators for various skin conditions (4).

## Case report

### Patient

**Figure 1:**
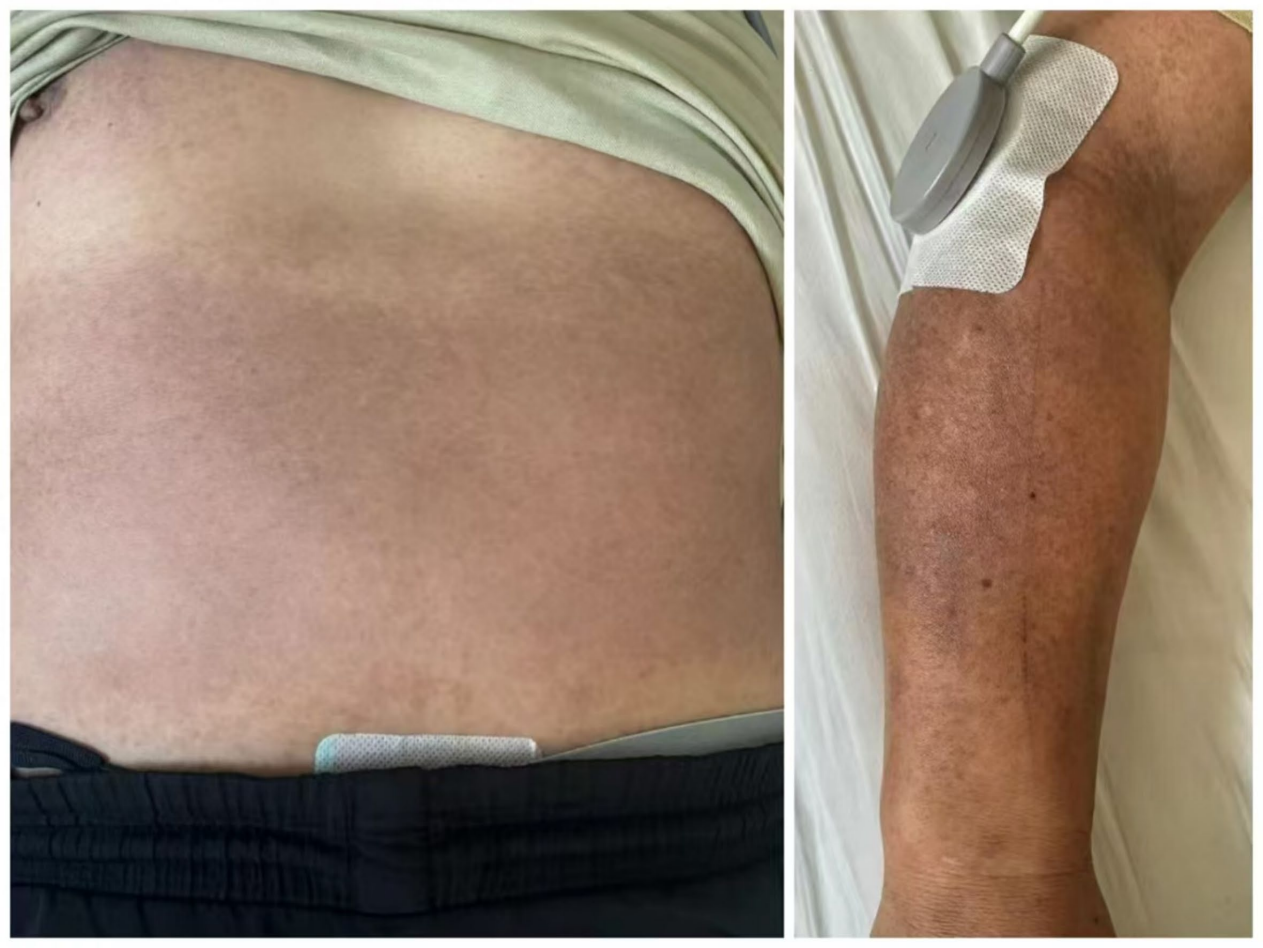
Skin condition of the patient prior to treatment.

A 45-year-old man with no significant medical history and no allergies was diagnosed with COVID-19 in early 2022. One week after recovering from the acute phase, he developed pronounced hyperpigmentation on the chest, abdomen, and both upper limbs. The lesions were asymptomatic except for occasional tingling, and muscle strength remained normal. Over the subsequent 2 years, he underwent continuous treatment with topical depigmenting agents (e.g., compound arbutin cream, retinoids) and oral antioxidants (e.g., vitamins C and E) without appreciable improvement. Neither the lesion size nor the tingling sensation abated. At presentation, examination revealed dark purple-red maculopapular lesions and marked hyperpigmentation on the chest, abdomen, and both upper limbs (Figure 1). A PGA evaluation was conducted, resulting in a score of 3 (moderate pigmentation). The patient’s self-assessment using PGA (5) indicated a severe condition, accompanied by expressed concern. Additionally, a VAS (6) pain score of 3 was recorded.

### Diagnostic process

During his medical visits, the patient received several diagnoses, including confluent and reticulated papillomatosis, atopic dermatitis, connective tissue disease, pigmented lichen planus, and impaired barrier function. Cardiac enzyme levels, blood tests, antinuclear antibody profiles, and a full series of humoral vaccinations were refined, all of which showed no abnormal results. Pathology examination revealed perivascular lymphocytic infiltration and scattered melanophages in the superficial dermis, an increase in melanin granules at the dermal-epidermal junction, and no increase in mast cell count as determined by CD117.

### Treatment history

Despite receiving several Western treatments, such as laser therapy, topical corticosteroids, and light therapy, the hyperpigmentation. The patient then requested Complementary and Alternative Therapies, so we performed acupuncture therapy with sterile stainless steel needles. Acupuncture points (7) included: Xuehai (SP10), Yanglingquan (GB34), Hegu (L14), Sanyinjiao (SP6), and Geshu (BL17) (Table 1). The treatment was given once a day for 30 min. The treatment lasted for 1 week.

**Table 1:** Localization and manipulation of acupuncture points.

Acupuncture point	Acupuncture point locations	Needle angle	Needle depth
Xuehai (SP10)	On the anteromedial aspect of the thigh, on the bulge of the vastus medialis muscle, 2 B-cun superior to the medial end of the base of the patella	Vertical	20–25 mm
Yanglingquan (GB34)	On the fibular aspect of the leg, in the depression anterior and distal to the head of the fibula	Vertical	20–25 mm
Hegu (L14)	On the dorsum of the hand, radial to the midpoint of the second metacarpal bone	Vertical	17–22 mm
Geshu (BL17)	In the upper back region, at the same level as the inferior border of the spinous process of the seventh thoracic vertebra (T7), 1.5 B-cun lateral to the posterior median line	45° angle toward the spine	20–22 mm
Sanyinjiao (SP6)	On the tibial aspect of the leg, posterior to the medial border of the tibia, 3 B-cun superior to the prominence of the medial malleolus	Vertical	20–25 mm

All points are WHO standards (7).

### Outcome

**Figure 2:**
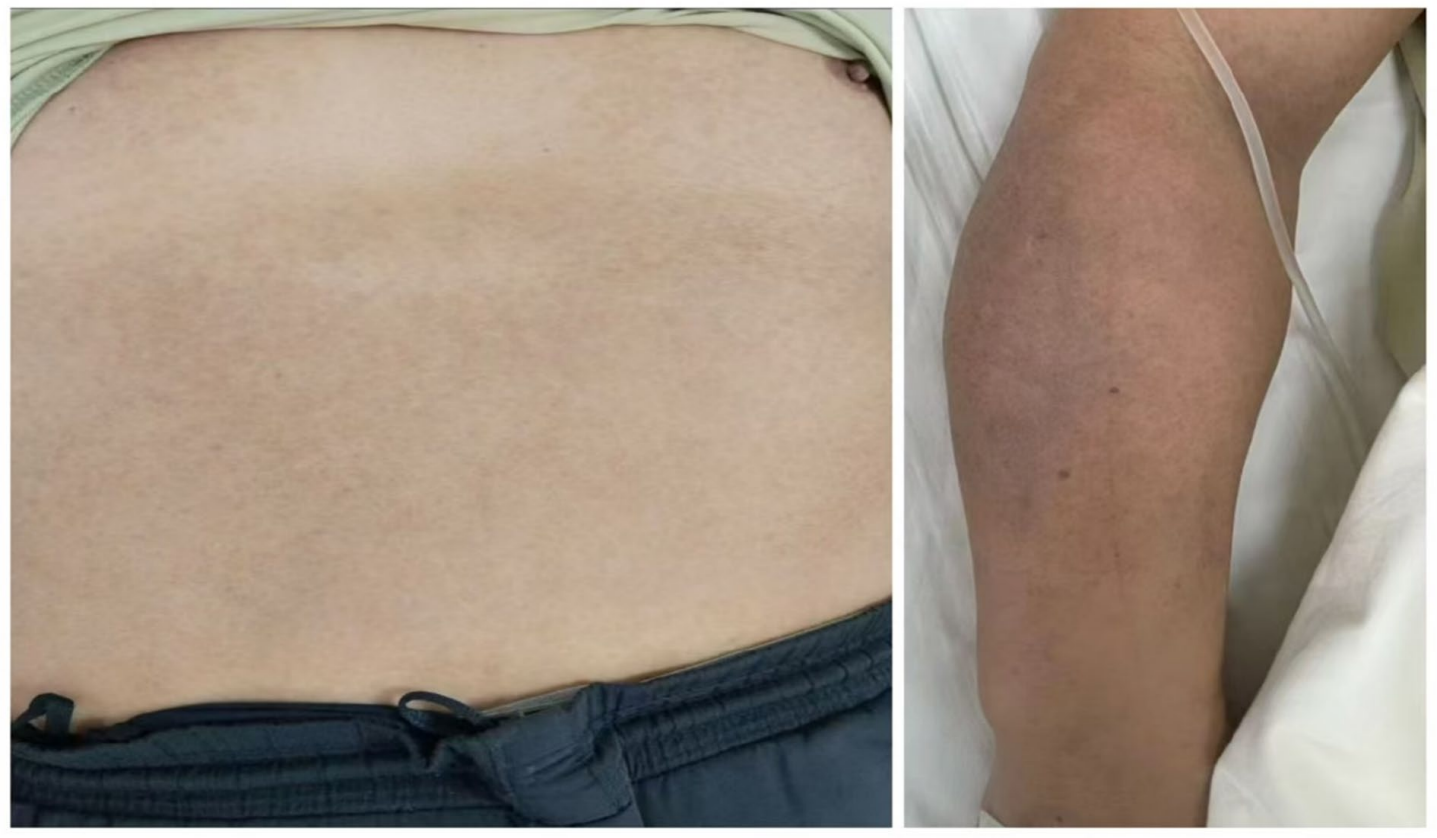
Skin condition of the patient following treatment.

After 1 week of acupuncture, the patient’s skin pigmentation showed significant improvement. The hyperpigmented areas on the anterior chest, abdomen, and upper limbs had lightened considerably, and the patient reported an improved skin texture with reduced discomfort. No adverse reactions were observed (Figure 2). Post-treatment, a reduction in lesion size, lightening of pigmentation, and decreased pain were observed. The GAIS score was 2 (“Much improved”), indicating a substantial reduction in pigmentation. PGA scores decreased from 3 to 1, and VAS pain scores reduced from 3 to 1. Unfortunately, the follow-up was unsuccessful due to invalid contact information.

## Discussion

We present the case of a patient with skin manifestations following infection with the novel coronavirus. After receiving the results of complementary and pathological examinations, a primary diagnosis of post-COVID hyperpigmentation was made. The patient received complementary therapies, including acupuncture, and her symptoms improved significantly over time.

We excluded the previous diagnosis based on ancillary tests and pathological findings despite similarities in the outward manifestations and symptoms of the initially diagnosed conditions. Connective tissue disease, which can cause skin lesions resembling a similar rash, did not present with autoimmune system changes in addition to the skin changes. Confluent and reticulated papillomatosis is characterized by the appearance of gray-brown papules on the skin, with a reticular or irregular pattern that may affect the entire body. Although its external manifestations are similar to those of our patient, the typical histopathological features of the disease were not observed. Pigmented lichen planus, while also resembling this case, often presents with abnormal pathological findings and has not been shown to cause this type of dermatosis following COVID-19. Atopic dermatitis can be excluded based on external symptoms and ancillary tests.

Although GAIS (8) and PGA (5) were not specifically designed to quantify pigmentation, they capture subjective and objective changes in related dermatological conditions. GAIS is suitable for assessing overall esthetic outcomes, while PGA allows clinicians to grade disease severity based on pigmentation changes. However, both instruments lack standardized pigmentation metrics and are inherently subjective. It is recommended that future randomized controlled trials incorporate additional objective measurements to enhance assessment accuracy.

Studies indicate that infection with COVID-19 can lead to nonspecific cutaneous changes (9), including rashes and other skin alterations. However, these changes often lack clear pathological features, making it difficult to classify as a specific skin disease. Potential mechanisms include the following:

1. Immune System Abnormalities: Following COVID-19 infection, the immune system may abnormally target skin cells, leading to an inflammatory response and symptoms such as rashes and itching (10).
2. Vascular Lesions: COVID-19 not only affects the respiratory system but can also cause endothelial damage to blood vessels, resulting in vasculitis, microthrombosis, and other complications. These pathological changes may contribute to nonspecific skin changes. Microthrombi, particularly in patients with prolonged COVID-19 sequelae, can impair blood flow to the skin, leading to color changes or rashes (11).

Although the exact mechanisms underlying acupuncture in the treatment of skin diseases remain unclear (12), evidence suggests that acupuncture stimulation can influence the hypothalamus–pituitary–adrenal (HPA) axis, the autonomic nervous system (ANS), and brain-derived neurotrophic factor (BDNF) (13). These systems are known to play critical roles in mediating stress responses, immune modulation, inflammation, and skin physiology, all of which are closely linked to skin conditions. Based on this, we hypothesize that acupuncture may affect skin pigmentation by modulating the HPA axis to increase cortisol levels and melanocyte activity, engaging the ANS to alter blood flow and inflammatory responses, and possibly impairing BDNF-mediated skin repair, in addition to inducing local microtrauma and inflammation at needle insertion sites. However, further studies are needed to validate this proposed mechanism. Moreover, functional magnetic resonance imaging (fMRI) studies have demonstrated that acupuncture can significantly activate the hypothalamus–limbic system (14), which is believed to contribute to skin diseases by promoting stress-related inflammation, immune dysregulation, and hormonal imbalances.

We selected Xuehai (SP10), Yanglingquan (GB34), Hegu (LI4), Geshu (BL17), and Sanyinjiao (SP6). In TCM, hyperpigmentation is attributed to blood stasis or blood heat; these acupoints are believed to enhance cutaneous circulation by promoting blood flow, resolving stasis, clearing heat, and cooling the blood. A meta-analysis of acupuncture for melasma identified SP10 as a commonly used point, particularly effective for blood-stasis–type pigmentation (15). LI4 is frequently employed to treat facial dermatoses such as freckles and melasma and has been shown to increase facial blood perfusion, correlating with pigmentation improvement (16). SP6 is among the most cited points for skin pigmentation treatments; one meta-analysis reported its use in eight studies, demonstrating significant effects (15).

Most acupuncture protocols for skin pigmentation prescribe two to three sessions per week. However, in early or severe cases, daily treatments may be advantageous. For example, a study on freckles recommended twice-weekly sessions, with daily treatments during the initial phase. In another melasma trial, acupuncture was administered over 2 months with unspecified frequency, though clinical practice often uses daily or alternate-day sessions. Standardized dermatosis protocols typically retain needles for 20–30 min; one study specifically reported a 30-min retention to optimize therapeutic stimulation.

A key limitation of this case report is the absence of quantitative data for objective efficacy assessment. Because the patient refused additional testing and was lost to follow-up, histopathological analysis and melanin content measurement were not feasible, limiting our ability to fully validate treatment effects. Nonetheless, detailed clinical observations and patient-reported outcomes indicate positive results. Furthermore, quantitative results from similar studies (15) corroborate our findings, supporting the efficacy of acupuncture in treating skin hyperpigmentation.

In this case, the patient developed cutaneous symptoms subsequent to SARS-CoV-2 infection, confirmed by a positive diagnostic test. The patient reported no medication use or allergen exposure during this period aside from the infection. While the temporal correlation implies a possible association between COVID-19 and skin manifestations, a single case report precludes causal inference. Further studies are necessary to exclude alternative factors and substantiate this relationship.

This case suggests that acupuncture may be effective for post-COVID-19 skin hyperpigmentation; however, given the inherent limitation of a single patient, further validation is required. We recommend that future RCTs include:

Sample size: 50–100 participants.
Control group: standard therapy or sham acupuncture.
Standardized efficacy assessments: skin colorimetry, high-resolution photography, and the Dermatology Life Quality Index (DLQI).
Follow-up schedule: evaluations at 3, 6, and 12 months to assess the durability of treatment effects.

The present case indicates that acupuncture may provide therapeutic benefit for post-COVID-19 cutaneous pigmentary abnormalities, particularly when conventional therapies prove ineffective. Given the suboptimal efficacy of standard interventions, acupuncture merits further investigation as an adjunctive treatment. However, as this conclusion is drawn from a single case report with inherently limited evidence, robust clinical trials are necessary to validate its efficacy and assess its generalizability.

## Data Availability

The original contributions presented in the study are included in the article/supplementary material, further inquiries can be directed to the corresponding authors.

## Footnotes

^1^ https://covid19.who.int/
